# Clinical decision making in the era of immunotherapy for high grade-glioma: report of four cases

**DOI:** 10.1186/s12885-018-4131-1

**Published:** 2018-03-01

**Authors:** Surabhi Ranjan, Martha Quezado, Nancy Garren, Lisa Boris, Christine Siegel, Osorio Lopes Abath Neto, Brett J. Theeler, Deric M. Park, Edjah Nduom, Kareem A. Zaghloul, Mark R. Gilbert, Jing Wu

**Affiliations:** 1 Neuro-Oncology Branch, Center for Cancer Research, National Cancer Institute, National Institute of Health, Bethesda, MD 20892 USA; 2 Laboratory of Pathology, National Cancer Institute, National Institutes of Health, Bethesda, MD 20892 USA; 3 Clinical Research Directorate/Clinical Monitoring Research Program, Leidos Biomedical Research, Inc., NCI Campus at Frederick, Frederick, MD 21702 USA; 4 Department of Neurology and John P. Murtha Cancer Center, Walter Reed National Military Medical Center, Bethesda, MD 20889 USA; 5 Surgical Neurology Branch, National Institute of Neurological Disorders and Stroke, National Institutes of Health, Bethesda, MD 20892 USA

**Keywords:** CTLA-4, Immune checkpoint inhibitors, Immunotherapy, Ipilimumab, iRANO, PD-1, Pseudoprogression, Nivolumab

## Abstract

**Background:**

Immune checkpoint inhibitors (ICPIs) are being investigated in clinical trials for patients with glioblastoma. While these therapies hold great promise, management of the patients receiving such treatment can be complicated due to the challenges in recognizing immune-related adverse events caused by checkpoint inhibitor treatment. Brain imaging changes that are the consequence of an inflammatory response may be misinterpreted as disease progression leading to inappropriate premature cessation of treatment. The aim of this study was to, by way of a series of cases, underscore the challenges in determining the nature of contrast-enhancing masses that develop during the treatment of patients with glioblastoma treated with ICPIs.

**Case presentation:**

We reviewed the clinical course and management of 4 patients on ICPIs who developed signs of tumor progression on imaging. These findings were examined in the context of Immunotherapy Response Assessment in Neuro-Oncology (iRANO) guidelines. Although all 4 patients had very similar imaging findings, 2 of the 4 patients were later found to have intense inflammatory changes (pseudoprogression) by pathologic examination.

**Conclusions:**

A high index of suspicion for pseudoprogression needs to be maintained when a patient with brain tumor on immunotherapy presents with worsening in an area of a pre-existing tumor or a new lesion in brain. Our findings strongly suggest that pathological diagnosis remains the gold standard for distinguishing tumor progression from pseudoprogression in patients receiving immunotherapy. There is a large unmet need to develop reliable non-invasive imaging diagnostic techniques.

**Trial registration:**

ClinicalTrials.gov NCT02311920. Registered 8 December 2014.

## Background

Despite multimodality treatment approaches and ongoing research, glioblastoma remains a deadly cancer with a median overall survival of 11 to 14.6 months [1–3]. Clinical trials based on cancer immunotherapy have come to the forefront of clinical research, delayed in part by concerns of immune privilege, that have proven to be mostly unfounded [4]. The success of immune checkpoint inhibitors (ICPIs) in various solid tumors like advanced melanoma, non-small cell lung cancer and renal cell cancer, has stimulated interest in testing these agents in glioblastoma [5–7].

In order to survive, tumor cells evade the body’s immune system by dysregulating immune checkpoints by the overexpression of immunosuppressive surface ligands [8, 9]. A number of immune checkpoint pathways have been successfully exploited in immune therapies. The early studies of checkpoint modulation have predominantly focused on cytotoxic lymphocyte-associated protein-4 (CTLA-4) and programmed cell death-1 (PD-1). CTLA-4 is a potent co-inhibitory ligand expressed only on activated T cells, which inhibits early steps of T-cell activation [10]. Similar to CTLA-4, PD-1 activation inhibits T-cell activation, but typically at later stages and at local sites of inflammation [4]. Likewise, interaction between PD-1 and one of its ligands, PD-L1, delivers a co-inhibitory signal causing T-cell dysfunction [11]. PD-L1 expression has been found in glioblastomas, however studies have reported a wide variability in PD-L1 expression from a modest expression of 2.8% [12] to diffuse staining in 88% of glioblastomas [13]. The presence of tumor-infiltrating lymphocytes with PD-1 expression has also been detected in glioblastoma specimens [13]. With the promise of seeing a benefit like other solid tumors, immune checkpoint inhibitors nivolumab and pembrolizumab (anti PD-1); ipilimumab (anti CTLA-4) and MEDI4736 (anti PD-L1) are currently being investigated in glioblastoma clinical trials.

The use of ICPIs in treatment of advanced tumor is challenging due to the toxicities caused by disinhibition of the immune system, now called immune-related adverse events (irAEs) [14]. Although autoimmune and inflammatory conditions in the central and peripheral nervous system can occur, such as Guillan-Barre, autoimmune encephalitis, hypophysitis and transverse myelitis; these are quite uncommon. However, an immune reaction in the tumor bed is thought to be quite common and is a major diagnostic challenge as the imaging appearance emulates tumor growth with increased T2-FLAIR changes and increased T1-contrast enhancement, hence the term pseudoprogression [15, 16]. In recognition of the complexities related to evaluating brain tumor imaging with immunotherapy, the Immunotherapy Response Assessment in Neuro-Oncology (iRANO) criteria were recently created stating that close observation with serial imaging is appropriate for patients with imaging worsening and stable neurologic function within the first 6 months of treatment [17]. After 6 months, new or continued worsening should be deemed tumor progression.

**Fig. 1:**
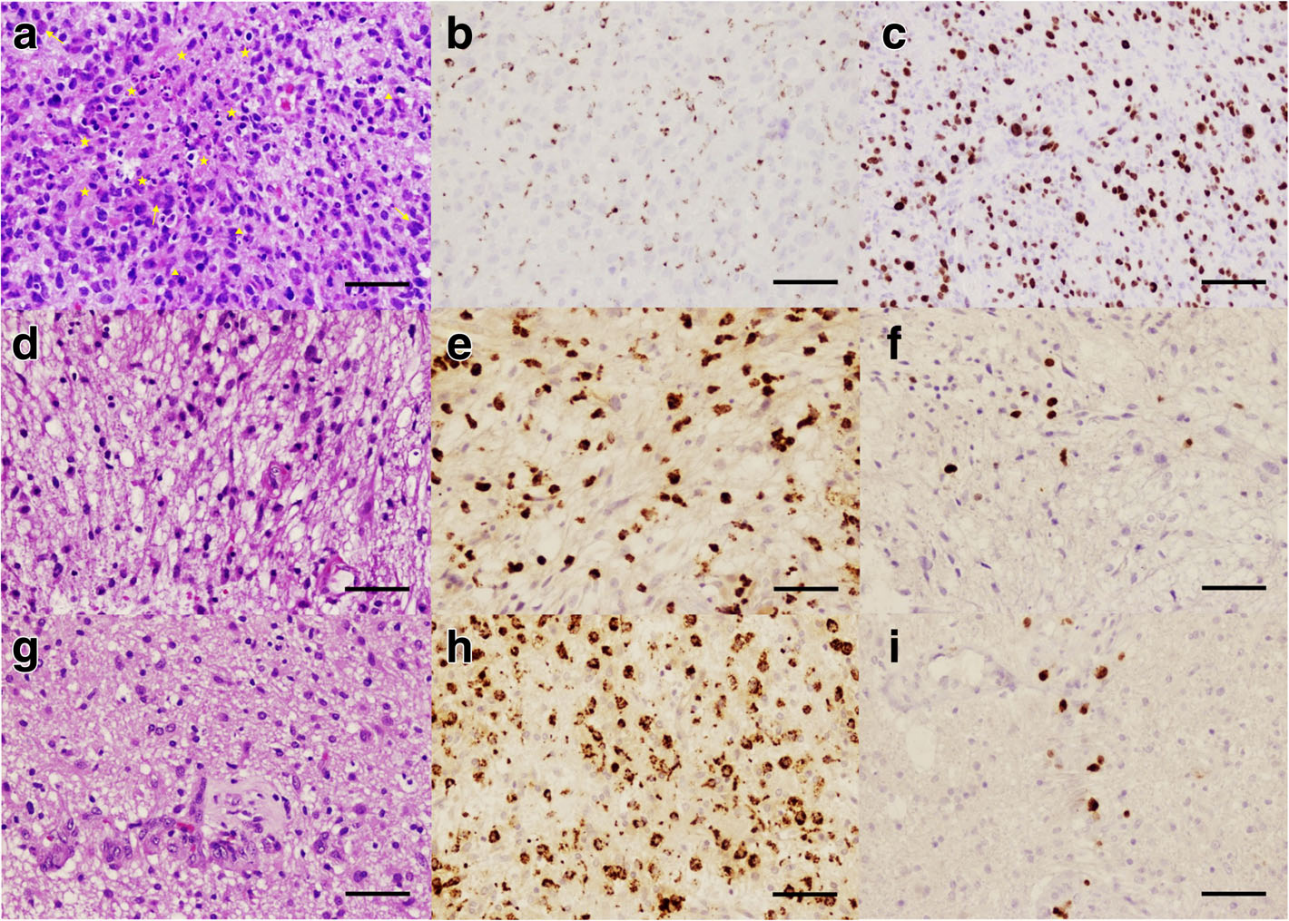
Tumor histology of patient P1. At initial diagnosis, H&E stain (**a**, 200X) shows a high grade glioma with increased cellularity, pleomorphic tumor cells (arrows), increased mitotic figures (arrowheads) and areas of necrosis (stars). Histiocytes are minimal in number as stained by KP-1 (**b**, 200X), and a high proliferative rate is detected by MIB-1 stain (**c**, 200X). At 3.5 months after initiating nivolumab treatment, a tumor biopsy shows on H&E stain (D, 200X) a much less cellular lesion with fewer atypical cells and marked histiocytic infiltration highlighted by KP-1 (**e**, 200X), suggesting reactive changes. MIB-1 stain (**f**, 200X) shows a much lower proliferative rate index. At 7 months, a new biopsy shows similar findings: on H&E (**g**, 200X) there is some increase in cellularity and cell atypia, still much less than prior to treatment. KP-1 stain (**h**, 200X) highlights a large number of histiocytes, and MIB-1 (**i**, 200X) continues to demonstrate a low proliferative rate index. Scale bar for all panels measures 150 μm

**Fig. 2:**
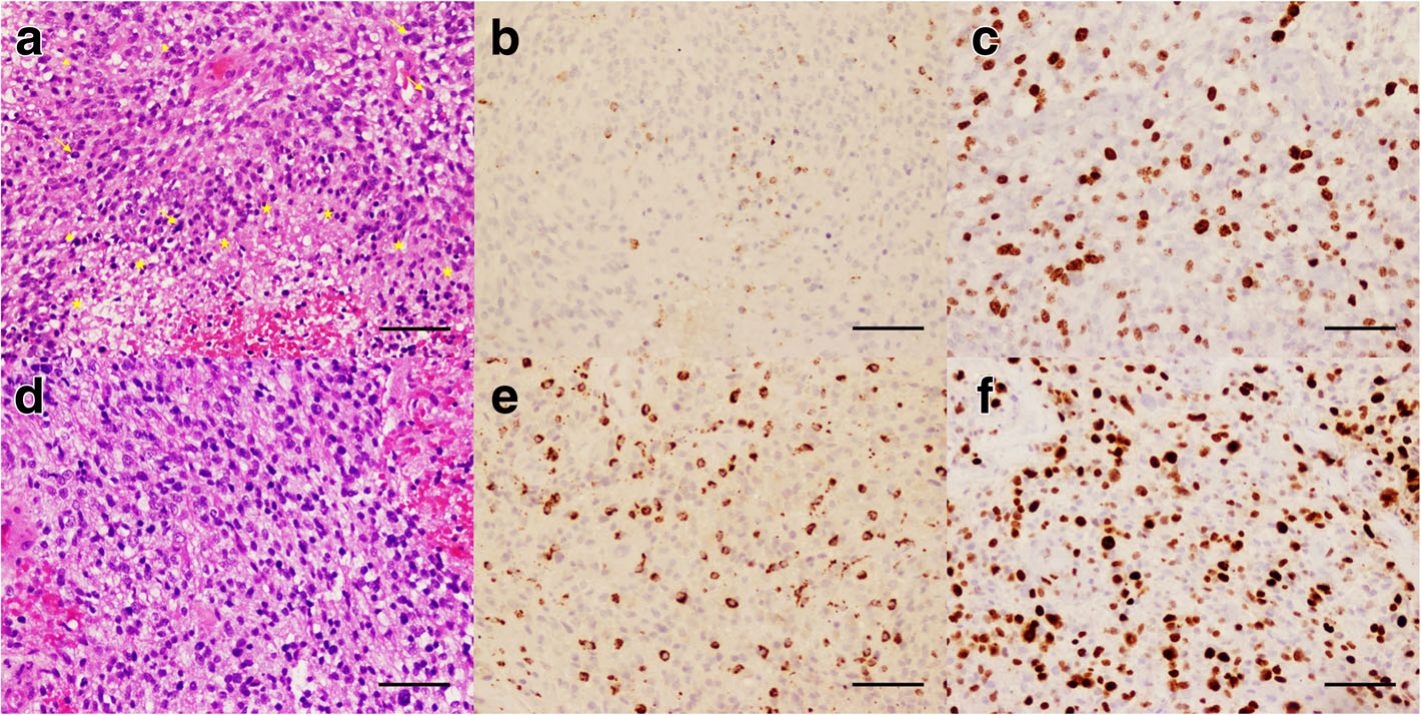
Tumor histology of patient P3. At initial diagnosis, H&E stain (**a**, 200X) reveals a high-grade glioma with pleomorphic tumor cells (arrows), increased mitotic figures (arrowheads), and areas of necrosis (stars). Histiocytes are minimal in number as stained by KP-1 (**b**, 200X). MIB-1 shows a high proliferative rate index (**c**, 200X). At 8.5 months after the initiation of ipilimumab, a new biopsy of the tumor still shows on H&E (**d**, 200X) a high-grade glial neoplasm with increased cellularity and mitotic figures. Reactive changes are present and abundant histiocytes are identified with KP-1 stain (**e**, 200X). MIB-1 demonstrates a high proliferative rate index, up to 40% in some areas. Scale bar for all panels measures 150 μm

Here we present a series of 4 patients with glioblastoma receiving ICPIs under our ongoing phase I clinical trial of ipilimumab, nivolumab and the combination in patients with newly diagnosed glioblastoma (NCT02311920), who developed a new brain lesion or had a radiographic worsening of pre-existing brain tumor. This clinical trial was approved by the institutional review board. All patients had signed written informed consents for participating in the trial and provided written consents for publication. All 4 patients underwent a gross total resection (GTR) of their primary glioblastoma followed by 6 weeks of radiation with concurrent temozolomide (TMZ) chemotherapy. Patients were enrolled in the study at the 4-week follow-up after chemoradiation and were randomized either to nivolumab or ipilimumab arm. All patients received adjuvant TMZ. The initial diagnosis of glioblastoma was confirmed at the Laboratory of Pathology, NCI (Figs. 1a, b, c and 2a, b, c). These cases underscore the challenges associated with interpreting imaging findings and applying iRANO guidelines in the clinical management of patients receiving ICPI therapy [17].

## Case presentation

### CARE guidelines were followed in reporting these 4 cases.

#### Patient 1

**Fig. 3:**
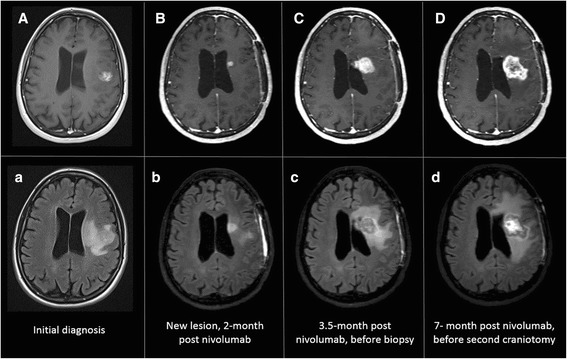
MRI images of P1 at initial diagnosis (**A** and **a**), at 2 months of initiation of nivolumab when a new enhancing lesion was noted in the centrum semiovale of the left frontal lobe (**B** and **b**), at 3.5 months of initiation of nivolumab prior to biopsy showing an increase in size of the enhancing lesion and mass effect on the left lateral ventricle (**C** and **c**) and at 7 months of initiation of nivolumab prior to second craniotomy illustrating a continued increase in size of the enhancing left frontal lesion and left frontal edema (**D** and **d**)

A 51-year-old woman (P1), who was previously healthy, presented with chief complaints of a 4-week period of speech difficulties. Cranial MRI revealed a left frontal mass which was enhanced with gadolinium (Fig. 3, A and a). After the surgery and concurrent chemoradiation therapy, she was enrolled to the study and randomized to receive nivolumab with TMZ. Two months after the initiation of nivolumab, an asymptomatic lesion was noted in the centrum semiovale of her left frontal lobe adjacent to the lateral ventricle (Fig. 3, B and b). This lesion was distant from her original tumor in the left frontal lobe and showed increased T1-contrast enhancement and abnormally elevated perfusion (not shown), which appeared suspicious for a recurrent high-grade glioma. However, she was clinically stable with a mild baseline expressive aphasia and treatment with TMZ and nivolumab was continued. The left frontal lesion continued to enlarge with mass effect on lateral ventricle due to increased vasogenic edema (Fig. 3, C and c). A biopsy was finally performed 3.5 months after trial enrollment; pathological exam revealed few atypical cells and marked lymphohistiocytic infiltration (Fig. 1, d and e), suggesting reactive changes. MIB1 index was very low and highest foci corresponded to vascular endothelial proliferation (Fig. 1f). Without evidence of active tumor progression, she was continued the therapies as per protocol.

Despite administration of dexamethasone, the brain MRI showed a gradual increase in size of the left frontal lesion with increased contrast enhancement and increased blood perfusion (Fig. 3D and d). Worsening expressive aphasia, right sided weakness and headaches with prompted resection of the left frontal lesion, 7 months after the initiation of nivolumab. Pathologic analysis revealed increased cellularity, cell atypia, glomeruloid vascular proliferation, vascular wall hyalinization and geographic necrosis (Fig. 1g). Many macrophages and lymphoid cells were identified (Fig. 1h). Like previous biopsy, mitotic figures were scant, and the MIB1 index was 3% (Fig. 1i). These findings were consistent with a brisk inflammatory reaction, treatment effect and residual tumor, demonstrating a diagnosis of immunotherapy-related pseudoprogression. Dexamethasone treatment improved her expressive aphasia and right-sided motor strength. One year later there has been no further growth of the mass lesion.

#### Patient 2

**Fig. 4:**
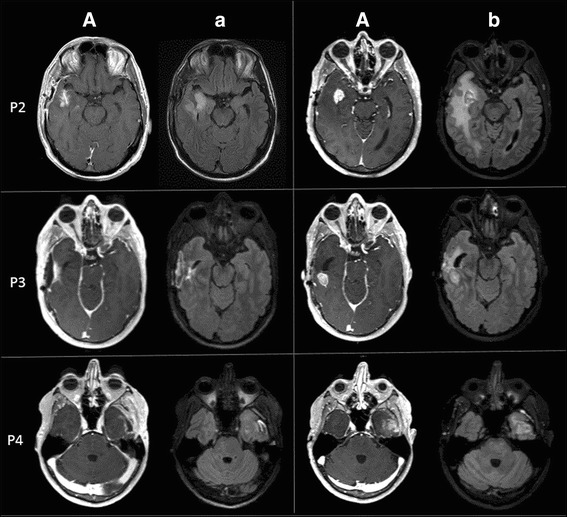
MRI images of the patients P2, P3 and P4 at 4 weeks post-chemoradiation (**A** and **a**) and at the time of biopsy or resection (**B** and **b**)

A 63-year-old man (P2) presented with intermittent receptive aphasia and was found to have a contrast-enhancing lesion in the right temporal lobe. He underwent a GTR followed by 6 weeks of concurrent radiation and chemotherapy (Fig. 4; P2, A and a). He was randomized to receive nivolumab and TMZ. Eight weeks after the commencement of nivolumab, he developed a new enhancing lesion within the right sylvian fissure with surrounding vasogenic edema at the site of initial tumor. Other than a minor focal seizure, he was otherwise clinically stable without new neurological signs. Therefore, this new lesion was favored to be inflammatory and his treatment was continued. He remained radiographically and clinically stable for the following 2 months. At a routine evaluation 5.5 months from the start of nivolumab, although he remained clinically asymptomatic, the right temporal lesion had increased in size and was associated with effacement of the adjacent cortical sulci and temporal horn of the right lateral ventricle. A sub-total resection of the contrast-enhancing right temporal lesion (Fig. 4; P2, B and b) and a right anterior temporal lobectomy were performed. Pathology of the contrast-enhancing region revealed an increased cellularity due to reactive changes with occasional atypical cells, increased numbers of histiocytes and microglia and a few lymphocytes with no evidence of active recurrent glioma and a MIB1 index of 3–5%. A small section of the right temporal lobe which was obtained from the non-contrast-enhancing part showed a focus of recurrent or residual glioma, occasional mitosis with reactive changes including the presence of perivascular and scattered T lymphocytes and infiltrating macrophages. However, when the rest of the right temporal lobe was sampled, no evidence of active or recurrent glioma was seen and the MIB1 was very low at 2–3%, concluding that the mass was comprised of inflammatory and reactive changes. He was diagnosed with immunotherapy-related pseudoprogression. He is currently at 10 months from initial diagnosis and continues on nivolumab infusions per the clinical trial.

#### Patient 3

A 47-year-old man (P3) presented with headaches, nausea and vomiting. Cranial MRI revealed a necrotic enhancing mass in the right temporal lobe with a slight midline shift and uncal herniation. He underwent a GTR, concurrent radiation and chemotherapy (Fig. 4; P3, A and a) followed by 6 cycles of adjuvant TMZ and ipilimumab without complication. Five-and-a-half months after the start of ipilimumab, he developed two small enhancing lesions in the right anterior and posterior temporal lobe surrounding the resection cavity. The patient remained asymptomatic, however both lesions continued to increase in size on MRI despite a steroid trial (Fig. 4; P3, B and b). A second temporal craniotomy with resection of the mass was performed 8.5 months from the start of immunotherapy. The larger posterior temporal lesion demonstrated recurrent glioblastoma (Fig. 2; D, E and F) with hypercellularity, cell atypia, mitotic activity and focal pseudopalisading necrosis. MIB1 index was up to 40%. The anterior temporal lesion showed mild hypercellularity and cellular atypia with only a few MIB1 positive cells, consistent with recurrent glioblastoma. This tumor progression mandated that the patient be taken off the study treatment. Patient was treated with concurrent radiation therapy with nivolumab off the protocol one month after the surgery. Nivolumab was chosen as an off-label salvage treatment, because its mechanism of action is distinct from ipilimumab and based upon our experience with patients P1 and P2. Interestingly, a similar worsened imaging finding was seen on the brain MRI 4.5 months after the nivolumab treatment. Pathologic exam revealed all inflammatory cells (indicating immunotherapy-related pseudoprogression) and nivolumab was continued. At 19 months from the initial diagnosis, he is currently stable.

#### Patient 4

A 47-year-old man (P4) presented with headaches and expressive aphasia. Cranial MRI revealed an irregular contrast-enhancing mass in the left temporal lobe. After a GTR and concurrent radiation therapy and chemotherapy (Fig. 4; P4, A and a), he was randomized to receive ipilimumab and TMZ. He tolerated the regimen well except for the development of inflammatory colitis controlled with methylprednisolone. Eight-and-a-half months after the initiation of immunotherapy, he developed an enhancing nodule in the left temporal lobe (Fig. 4; P4, B and b). At this time, he was complaining of focal seizures manifesting as transient right leg paraesthesias in setting of discontinuation of seizure medication. The left temporal resection done 2 weeks later demonstrating hypercellularity, increased mitosis, vascular proliferation, pseudopalisading necrosis consistent with active glioblastoma with the highest MIB1 of 15%. He was removed from the study and treated with re-radiation with low-dose bevacizumab support, and off-label nivolumab. He is now 21-month status post the intial diagnosis of glioblastoma and clinically stable.

## Discussion and conclusions

The clinical history of all 4 patients is summarized in Table 1, in accordance with CARE guidelines. This case-series highlights the diagnostic conundrum of managing glioblastoma patients with imaging changes on standard MRI protocols where, months after immunotherapy, diagnostic imaging shows an area of increased contrast enhancement and T2/FLAIR changes, often with worsening neurologic function. In our treatment setting, with the upfront initiation of both chemoradiotherapy and immunotherapy, clinical and imaging findings are further complicated by the fact that each of these treatments can induce pseudoprogression. Conventional MRI cannot be used to reliably differentiate tumor progression from pseudoprogression [18–20]. A study showed that the subependymal enhancement may predict true tumor progression rather than pseudoprogression from radiochemotherapy [21], whereas another did not [19]. The periventricular white matter is a favored site for pseudoprogression due to radiochemotherapy [22], and interestingly this is also seen in patients P1 and P2. The predilection of pseudoprogression in the periventricular white matter may be because of a paucity of blood supply in the region, which leads to a higher vulnerability to post-radiation vasculopathy and ischemia [22]. The conventional MRI pattern of pseudoprogression after immunotherapy has not been fully characterized. A report of 2 cases of intralesional immunotherapy in glioblastoma shows immunotherapy-related pseudoprogression in the perilesional and periventricular areas [23], like our patients P1 and P2.

**Table 1:** Timeline of care for all patients

	Patient 1	Patient 2	Patient 3	Patient 4
Initial diagnosis & intervention	- Left frontal glioblastoma - GTR - Radiation + concurrent TMZ - Nivolumab	- Right temporal glioblastoma - GTR - Radiation + concurrent TMZ - Nivolumab	- Right temporal glioblastoma - GTR - Radiation + concurrent TMZ - Ipilimumab	- Left temporal glioblastoma - GTR - Radiation + concurrent TMZ - Ipilimumab
Changes on MRI brain #1 & clinical symptoms	- New enhancing lesion in the left frontal lobe distal to initial tumor - Clinically stable	- New right temporal lesion at the site of intial resection - Focal seizure	- 2 lesions in right temporal lobe near resection cavity - Asymptomatic	- New enhancing lesion in left temporal lobe - Focal seizure
Time from initiation of ICPI #1	- 2 months	- 2 months	- 5.5 months	- 8.5 months
Intervention #1	- Biospy at 3.5 months of ICPI, when aphasia worse	- Resection at 5.5 months of ICPI, when lesion increased in size - Clinically stable	- Resection at 8.5 months of ICPI, when lesion increased in size despite dexamethasone	- Resection at 9 months from ICPI
Pathology #1	- Pseudoprogression	- Pseudoprogression	- Tumor progression	- Tumor progression
Treatment	- Continued nivolumab	- Continued nivolumab	- Discontinued ipilimumab - Concurrent radiation + off-label nivolumab	- Discontinued ipilimumab - Re-radiation, low-dose bevacizumab and off-label nivolumab
Changes on MRI brain #2 & clinical symptoms	- Increased size of the left frontal lesion - Worsened aphasia, right-sided weakness and headaches		- Increased size of right temporal lesion	
Time from initiation of ICPI #2			- 4.5 months from nivolumab initiation	
Intervention #2	- Trial of dexamethasone - Resection at 7 months of ICPI		- Resection at 5 months from nivolumab initiation	
Pathology #2	Pseudoprogression		Pseudoprogression	
Follow-up	- Stable at 12 months from diagnosis	- On nivolumab at 10 months from diagnosis	- Continued nivolumab, 19 months from diagnosis	- Continued nivolumab, 21 months from diagnosis

Advanced MR techniques and metabolic imaging provide helpful objective data in distinguishing tumor progression from immunotherapy-related pseudoprogression [23–26].

However, the utility of these imaging approaches hasn’t been validated for a definitive diagnosis of tumor growth versus a treatment-related region of inflammation. The four cases described in this report required surgical procedures to determine the cause of the worsening clinical findings. The two patients who were noted to have new or worsening brain lesions which first appeared at 8 weeks of initiation of immunotherapy, were found to be pseudoprogression by pathologic evaluation after removal of the tumor mass. Conversely, the two other tumor patients had worsening of brain lesions at 5.5 and 8.5 months after initiation of immunotherapy and these were confirmed to be tumor progression after resection and pathologic examination. The early development of inflammatory changes may be the result of adding immunotherapy to concurrent chemotherapy and radiation that may synergistically enhance multiple cell death pathways, leading to exacerbation of pseudoprogression [20]. Patient P3 with confirmed tumor progression was treated with a repeat course of radiation followed by treatment with nivolumab. Resection of a growing mass 5 months after the salvage regimen demonstrated only an inflammatory reaction. In this patient the development of pseudoprogression beyond 3 months after radiation is likely to be attributed to immunotherapy, rather than radiation. Conventional MR imaging was unhelpful in diagnosis. Though not used in our case, MR perfusion-weighted imaging, MR diffusion-weighted imaging, MR spectroscopy (MRS) and amino acid PET may be used to differentiate immunotherapy-related pseudoprogression from recurrent glioblastoma. Relative CBV in contrast-enhancing lesion is higher in patients with tumor progression in comparison to patients with immunotherapy-related pseudoprogression [25, 26]. Minimum ADC value is lower in progressive tumor patients as compared to the patients who have stable disease [26]. In two patients with recurrent glioblastoma treated with intratumoral immuntherapy, the areas of contrast-enhancement on conventional MRI did not exhibit corresponding high choline concentrations on MRS, as it would be expected for tumor progression [23]. PET using radiolabeled amino acids such as O-(2-[18F] fluoroethyl)-L-tyrosine (FET) may also have future application in distinguishing tumor progression from immunotherapy-related pseudoprogression. A retrospective series studied 5 patients with melanoma brain metastases treated with immunotherapy [24]. Of this 1 patient was classified as pseudoprogression while 4 had tumor progression. The maximum tumor-to-brain ratio of FET uptake values were considerably higher in cases of tumor progression as compared to the patient with immunotherapy-related pseudoprogression.

Interestingly, all three cases of pseudoprogression (P1, P2 and P3 after salvage treatment) appeared after initiation of nivolumab. PD-1 and CTLA-4 inhibition have their own unique mechanisms of action. CTLA-4 has both cell-intrinsic (CTLA-4 on effector cells) and cell-extrinsic (CTLA-4 on T cells) activity and is antigen non-specific in contrast to PD-1 which is primarily antigen-specific and cell-intrinsic [27]. Data from glioblastoma clinical trials comparing the efficacy and adverse-effect profiles of nivolumab versus ipilimumab has not matured yet for the authors to make a definite statement that pseudoprogression is more likely with nivolumab.

### The iRANO guidelines define a 6-month window of immunotherapy-related pseudoprogression

The unique challenges associated with interpretation of new lesions or worsening of pre-existing lesions in patients getting treated with immunotherapy have led to the development of iRANO guidelines in 2015 [17]. The iRANO guidelines are modifications of the pre-existing Response Assessment in Neuro-Oncology (RANO) criteria to allow for longitudinal follow-up before a diagnosis of progressive disease is made in patients receiving immunotherapy. The RANO criteria, first published in 2010, provide guidance for considering pseudoprogression after concurrent radiation and chemotherapy. The guidelines state that a diagnosis of progressive disease should not be made within 12 weeks of completion of concomitant chemoradiation, unless there is new enhancement outside the radiation field or confirmation of active tumor from pathologic examination. Immunotherapy is expected to create an inflammatory response in areas of tumor involvement, consequently an exacerbation of pseudoprogression is anticipated and has been described in anecdotal case reports [28–30]. Therefore, the iRANO guidelines widen the window of pseudoprogression to 6 months, justified by the anticipated delay in the induction of an immune response, particularly when immunotherapy is administered after the completion of the chemoradiation. The iRANO guidelines underscore the variable nature of pseudoprogression with immunotherapy and therefore, mandate that radiographic progression be confirmed on follow-up imaging ideally at 12 weeks after initial scan showing disease progression, unless there is clinical deterioration. Unlike RANO criteria, given the possibility of immunotherapy causing a remote inflammatory reaction, the appearance of a new distal lesion may not automatically lead to a diagnosis of tumor progression.

### Tissue diagnosis is required to diagnose pseudoprogression with immunotherapies

The iRANO guidelines anticipate that there will be an increased incidence of pseudoprogression with immunotherapy and provide response (and failure) assessment guidelines for clinical trials and clinical care. However, the pseudoprogression timeframe outlined in these guidelines should be prospectively validated to determine this with better accuracy. Furthermore, as we noted from our patient series, asymptomatic lesions may not always be pseudoprogression and true disease progression may not always be symptomatic. Corticosteroids may be initiated to help reverse the mass associated edema, but it has not been established whether inflammatory lesions are more likely to respond than true progressive tumor. Currently, most clinical trials testing immunotherapy in patients with brain tumors state that if clinical examination is stable, immunotherapy can be continued. Conversely, a large increase in the size of the lesions or clinical deterioration would warrant treatment discontinuation unless a biopsy or craniotomy confirms pseudoprogression. Our findings would support tumor sampling or preferably, resection to help establish the diagnosis.

However, the interpretation of the tissue obtained from biopsy or resection may be challenging. Small biopsy samples may be misleading because of sampling error and even with extensive resection, the distinction between recurrent and residual tumor can be challenging. No formal criteria have been established to distinguish active, progressive tumor from residual and biologically inactive tumor. Most pathologists rely on tumor cell density, finding of mitotic figures and Ki-67 immunohistochemical staining demonstrating an elevated proliferative index in tumor cells. The finding of accumulated immune cells, including lymphocytes and macrophages, would be expected with pseudoprogression, but there are no established guidelines to help interpret these results. After obtaining a pathological diagnosis, further longitudinal confirmation with stable MRIs will cement the diagnosis of immunotherapy-related pseudoprogression.

A high index of suspicion should be maintained for the possibility of pseudoprogression, when patients with glioblastoma treated with immunotherapy develop a worsening of pre-existing tumor(s) or new brain lesions. Currently, clinical examination or advanced imaging techniques cannot accurately distinguish bona fide tumor progression from pseudoprogression. Until reliable non-invasive diagnostic techniques are developed, a pathologic diagnosis with an emphasis on tumor cell density, mitotic activity, Ki-67 immunohistochemical stains and the presence of immune cells, remains the gold standard for the diagnosis of pseudoprogression. The establishment of a pathological diagnostic criterion of immunotherapy-related pseudoprogression will be valuable.

## Abbreviations

CBV: Cerebral blood volume
CTLA-4: Cytotoxic lymphocyte-associated protein-4
ICPI: Immune checkpoint inhibitor
irAE: Immune-related adverse event
iRANO: Immunotherapy response assessment in neuro-oncology
MRI: Magnetic resonance imaging
MRS: Magnetic resonance spectroscopy
PD-1: Programmed cell death-1
PET: Positron emission tomography
RANO: Response assessment in neuro-oncology
TMZ: Temozolomide
